# High Expression of CpMAPK9 Increased Jasmonic Acid Levels Potentially Improving Root Rot Resistance in *Codonopsis pilosula*

**DOI:** 10.3390/genes17080890

**Published:** 2026-07-29

**Authors:** Jiahao Cao, Yufei Cheng, Yichuan Liang, Ao Li, Mingming Yang, Xiaotong Guo, Linlin Dong

**Affiliations:** 1 College of Horticulture, Ludong University, Yantai 264000, China; 2 Key Laboratory of Beijing for Identification and Safety Evaluation of Chinese Medicine, Institute of Chinese Materia Medica, China Academy of Chinese Medical Sciences, Beijing 100700, China; 3 State Key Laboratory of Chinese Medicine Modernization, Tianjin University of Traditional Chinese Medicine, Tianjin 301617, China

**Keywords:** *Codonopsis pilosula*, biotic stress, root rot, MAPK, hormone

## Abstract

Background: This study aimed to investigate the molecular mechanisms underlying the response of *Codonopsis pilosula* roots to *Fusarium oxysporum* infection, which causes root rot and significant economic losses, and to provide a theoretical basis for breeding resistant varieties and developing effective control strategies. Methods: Root samples of C. pilosula were inoculated with *F. oxysporum* and harvested across five distinct intervals (0, 6, 24, 72, and 120 h after inoculation). Transcriptome sequencing was performed on 15 samples, generating 171.65 GB of clean data. De novo assembly was used to construct unigenes, followed by functional annotation and differential expression analysis. In addition, *CpMAPK9* was transiently overexpressed in *C. pilosula* leaves to evaluate its role in jasmonic acid (JA) biosynthesis and disease resistance. Results: A total of 94,896 unigenes were obtained. Functional analysis showed that genes involved in hormone signal transduction, the MAPK signaling pathway, and plant–pathogen interactions were consistently activated in response to infection. Among the MAPK gene family, *CpMAPK9* showed significant expression changes. Transient overexpression of *CpMAPK9* significantly increased JA accumulation, indicating that *CpMAPK9* positively regulates JA biosynthesis and may enhance resistance to root rot. Conclusions: In conclusion, our findings indicate that CpMAPK9 actively modulates JA-mediated defense pathways in C. pilosula during *F. oxysporum* invasion. Our findings shed light on the intricate molecular networks that drive disease defense, offering valuable theoretical insights to guide subsequent crop improvement initiatives.

## 1. Introduction

*C. pilosula* Franch, a highly valued medicinal plant in China, is renowned for its dried roots, which are traditionally used to replenish vital energy (qi) and offer numerous health benefits. Such benefits include enhancing immunity, improving microcirculation, and supporting cardiovascular health [1]. However, the cultivation of *C. pilosula* is significantly challenged by various diseases and pests, with root rot being one of the most severe. Root rot, primarily caused by *Fusarium oxysporum*, leads to the cracking and rotting of the roots—the primary medicinal organs—resulting in substantial economic losses [2]. As a prominent member among the trio of primary soil-borne phytopathogens, *F. oxysporum* is notoriously difficult to control due to its prolonged survival in soil and resistance to conventional management strategies [3]. This pathogen has been extensively studied in crops such as tomatoes, apples, cucumbers, and *Panax notoginseng* [4,5,6,7], but the molecular mechanisms underlying *C. pilosula*’s response to *F. oxysporum* infection remain poorly understood.

Plant immune mechanisms represent a sophisticated and multi-layered process that activates resistance at the molecular level upon pathogen infection. This process encompasses three critical stages, namely signal perception, signal transduction, and the execution of defense responses [8]. At the core of plant immunity are two distinct but interconnected mechanisms: namely PTI (pattern-triggered immunity) and ETI (effector-triggered immunity). The onset of PTI occurs upon the sensing of pathogen-associated molecular patterns by surface-localized pattern recognition receptors (PRRs), which in turn activates a basal immune response [9]. Conversely, ETI is activated when intracellular resistance (R) genes recognize specific pathogen effectors, leading to a stronger and more specific defense response. R genes are considered key targets for breeding resistant plant varieties due to their ability to confer disease resistance through gene-for-gene interactions. In contrast, susceptibility (S) genes can aid pathogen colonization by attenuating host defense mechanisms, ultimately rendering plants more prone to disease [10]. Among the various signaling pathways involved in plant immunity, hormone signaling and mitogen-activated protein kinase (MAPK) cascades play pivotal roles. Hormonal signaling, especially through salicylic acid (SA), jasmonic acid (JA), and ethylene, plays a vital role in regulating plant responses to biotic stress. These pathways regulate the expression of defense-related genes and the production of antimicrobial compounds. Similarly, MAPK signaling cascades serve as central hubs for transmitting immune signals, integrating inputs from various receptors, and amplifying defense responses. Together, these pathways form a complex network that enables plants to mount effective defenses against a wide range of pathogens. Understanding these mechanisms is essential for developing strategies to enhance plant resistance and improve crop resilience in the face of pathogen threats.

When plants are exposed to external stimuli, the SA signaling pathway, JA signal-transduction pathway, and abscisic acid (ABA) signal-transduction pathway respond to stimuli accordingly. Subsequently, they regulate the expression levels of related genes and proteins [11]. ABA is a vital phytohormone regulating both vegetative development and environmental stress adaptation. When the environment is optimal, ABA is reduced to base levels, promoting optimal growth. When a plant experiences a non-optimal environment, ABA levels in the plant change in response to stress injury. ABA functions in organs by binding to intracellular receptors known as PYLs [12]. ABA binds the intracellular receptor PYLs to form complexes with PP2C, allowing the inhibition of SnRK2 protein kinase to be released through PP2C [13]. SnRK2 is then activated by autophosphorylation or by other protein kinases, such as Raf-like MAPKK kinases (MAPKKKs) [14].

The MAPK-level pathway is a conserved pathway of eukaryotic signal transduction [15]. MAPK activation is one of the earliest signal events after plants perceive invading pathogens [16]. MAPKs are activated by the upstream kinases, MAPK kinases (MAPKKs), as well as by the phosphorylation of one Thr and one Tyr residue in the Thr-x-Tyr activation motif of MAPKs. The MAPK cascade is an important signaling pathway that acts downstream of the sensor/receptor in eukaryotes and converts the signals generated by the sensor/receptor into cellular responses [17]. The plant MAPK cascade is essential for controlling plant growth and adaptation to diverse stress conditions, including pathogen attack, drought, osmotic imbalance, and ultraviolet radiation [18].

Here, we performed a global transcriptome analysis on *C. pilosula* infected by *F. oxysporum* in order to understand how plants respond to root rot and to examine the potential molecular mechanisms. The study focused on the MAPK gene family, identifying key genes, and exploring their functions, thereby laying a foundation for understanding the resistance mechanisms of *C. pilosula* to root rot. Additionally, the findings offer valuable insights for developing resistant *C. pilosula* cultivars.

## 2. Materials and Methods

### 2.1. Plant Materials and F. oxysporum Inoculation

Seedlings of *C. pilosula* were obtained from the Green Standardized Chinese Medicinal Crop Cultivation Base in Qingyuan Town. After treatment, the plants were placed in a greenhouse. They were irrigated with Hoagland nutrient formulation under a constant temperature of 25 °C and a 16 h photoperiod (16 h light/8 h dark). A relative humidity of 65–70% was continuously regulated throughout the cultivation.

*F. oxysporum* was isolated and saved in our laboratory, and the pathogen identity was confirmed [7]. *F. oxysporum* was cultured in liquid potato dextrose broth at 28 °C with shaking at 180 rpm for five days in darkness. The resulting conidial suspension was quantified with a hemocytometer. Each pot designated for inoculation received 250 mL of suspension containing (2 × 10^6^ conidia/mL), whereas control pots were treated with an equal volume of potato dextrose broth. Following an 8 h period, the seedlings were transplanted into soil and grown in a greenhouse environment. Three biological replicates were prepared for each time point, with each representing pooled root samples from three separate plants. Root samples were collected at 0, 6, 24, 72, and 120 h post-inoculation.

### 2.2. Measurement of Hormone Contents

*C. pilosula* roots were rapidly frozen in liquid nitrogen and pulverized. For each sample, a 0.5 g aliquot of the ground powder was subjected to extraction using 4 mL of a methanol:water:formic acid mixture (15:4:1, by volume). The mixture was then vortex-mixed over a 30 s period, followed by a 15 min sonication, and subsequently kept at 20 °C for another 20 min. Samples were centrifuged at 10,000× *g* and 4 °C for 10 min, after which the supernatants were transferred to fresh tubes. The residues underwent a second extraction under identical conditions, and the resulting extracts were pooled. The pooled extracts were vacuum-dried under a temperature of 45 °C, redissolved in 300 μL of methanol, and then subjected to another centrifugation at 10,000× *g* (4 °C) for 10 min. Finally, the clarified extracts were passed through 0.22-μm filters and placed in autosampler vials. The phytohormone profiles were determined using a UPLC-MS system (Xevo TQ-S; Waters, Wilmslow, UK), with three independent biological replicates analyzed for each sampling time point.

### 2.3. RNA Extraction and Sequencing

RNA was extracted from *C. pilosula* roots collected at different time points post-inoculation. Three biological replicates were collected for each time point. All samples were immediately placed in liquid nitrogen and ground to a fine powder. Total RNA was isolated using a TPIant RNA extraction kit (Bioteke, Beijing, China). Subsequently, Lianchuan Biotechnology Co., Ltd. (Hangzhou, China) carried out the RNA sequencing.

### 2.4. Bioinformatics Analysis

Adaptor sequences were removed using Cutadapt software (v3.4). The quality-filtered reads were mapped onto the reference genome with HISAT2 (v2.0.4). Transcript assembly for each sample was performed with StringTie. Based on the experimental strategy described previously with minor modifications [7], differentially expressed mRNAs were identified using the R package DESeq2 based on three biological replicates per time point, with a threshold of |log_2_FoldChange| ≥ 1 and adjusted q < 0.05. K-means clustering was carried out on the Metware platform. DEGs identified from pairwise comparisons between infected time points (6, 24, 72, and 120 h) and the control (0 h) were used for clustering analysis. Genes within each cluster or module underwent KEGG pathway enrichment and GO analysis using the OmicStudio tools.

### 2.5. Molecular Cloning and Leaf-Based Transient Upregulation of CpMAPK9

The *CpMAPK9* coding sequence was PCR-amplified, while the Super1300 plasmid was digested with XbaI and KpnI to generate a linearized vector. Subsequently, the amplified fragment was ligated with the digested vector using the TreliefTM SoSoo Cloning Kit (Tsingke, Beijing, China). To generate the expression strain, this recombinant vector was transformed into Agrobacterium tumefaciens strain GV3101 (Supplementary Figure S1). Bacterial suspensions were infiltrated into *C. pilosula* leaves under vacuum. Post-infiltration, the inoculated plants were cultured under a diurnal cycle of 25 °C/18 °C (light/dark) for three days. Subsequently, CpMAPK9 transcript abundance was quantified via quantitative real-time PCR, with three independent biological replicates included in the analysis.

### 2.6. qRT-PCR Analysis

Using a pre-chilled mortar and pestle, we ground the fresh plant tissues thoroughly in liquid nitrogen. Total RNA was then extracted using the Quick RNA Isolation Kit (Waryoung, Beijing, China), followed by reverse transcription into cDNA via the Fast Quant RT Kit (Tiangen, Beijing, China). Quantitative real-time PCR was conducted on a SLAN-96P Realtime PCR system (SLAN, Shanghai, China) with StarLighter SYBR Green qPCR Mix (Forever star, Beijing, China). Each target gene was examined using three independent biological samples, with three technical replicates for each sample. *CpiGAPDH* was used as the reference gene for normalizing gene expression levels in *C. pilosula* [19]. Gene expression fold changes were estimated according to the comparative threshold-cycle approach, using 2^−ΔΔCt^ [20]. The qRT-PCR primer sequences are provided in Supplementary Table S1.

### 2.7. Statistical Analysis

All statistical analyses were carried out in GraphPad Prism (v8.0) and R (v4.1.2). Two-group comparisons were performed via Student’s *t*-test, with significance thresholds of * *p* < 0.05 and ** *p* < 0.01. Data are shown as mean ± SD, with *n* = 3 biological replicates.

## 3. Results

### 3.1. Transcriptional Characterization of C. pilosula in Response to F. oxysporum Infection

One-year-old *C. pilosula* plants were selected as experimental materials and inoculated with *F. oxysporum* to study the infection process. At 72 h post-inoculation, typical disease lesions began to appear on the roots. By 120 h, the roots exhibited clear necrotic symptoms (Supplementary Figure S2), confirming successful infection by *F. oxysporum*.

To further investigate the resistance mechanism of *C. pilosula* against root rot, transcriptome analysis was performed on *C. pilosula* roots collected at five time points (0, 6, 24, 72, and 120 h) following *F. oxysporum* infection and a transcriptomic database of *C. pilosula* responses to *F. oxysporum* infection was established. A total of 658,298,922 clean reads were generated across 15 samples, including three biological replicates for each time point, and the contig N50 length was 1435 kb. The raw reads ranged from 34,084,348 to 56,218,658, and over 97.04% of reads were validated to clean reads. Q20% ranged from 97.84 to 98.29, and Q30% ranged from 93.69 to 94.4. Following assembly, a total of 94,896 genes (201 bp min length to 14,715 bp max length) were generated and the average GC content of the sequencing reads was 42.8% (Table 1). The sequencing quality and filtering results of *C. pilosula* transcriptome samples were good, confirming their viability for follow-up analysis.

PCA showed that 0 and 120 h samples were separated, and 6, 24, and 72 h samples were gathered. This finding suggested that from 6 h to 72 h, the plants were under similar biology processes. Meanwhile, repetitions of each time point were gathered, and samples were separated from one another at each time point (Supplementary Figure S1). FPKM (fragments per kilobase of transcript per million mapped reads) was used to quantify gene expression levels, which displayed a strong correlation among replicates, with Pearson’s correlation coefficients exceeding 0.81. Additionally, the expression of six selected DEGs was verified by qRT-PCR. The final qPCR data for the six DEGs were in accordance with the results of the transcriptome analysis (Supplementary Figure S3). Pearson analysis showed that qRT-PCR data of the six selected DEGs were significantly correlated with the RNA-Seq results (*p* < 0.05). These findings demonstrated the high quality and credibility of our RNA-seq data, justifying their application in subsequent investigations.

### 3.2. Analysis of the Differentially Expressed Genes Revealed Disease Resistance Pathways

**Figure 1:**
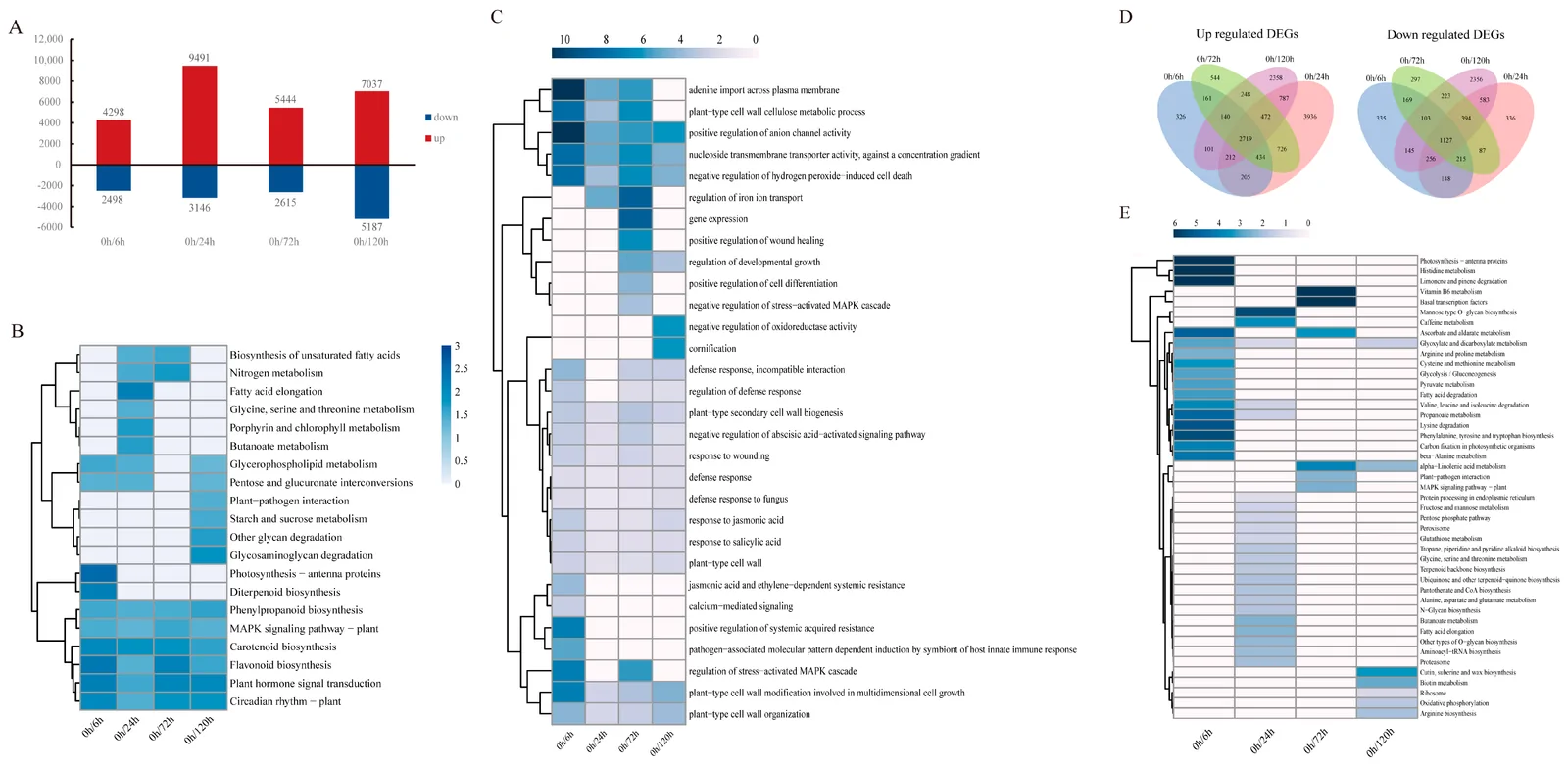
Overview of DEG statistics and functional enrichment during infection. (**A**) Number of upregulated and downregulated DEGs at different time points compared with 0 h. KEGG pathway (**B**) and GO term (**C**) enrichment of DEGs in pairwise comparisons. Z-score value of normalized −log10 (*p*-value) of each item was used to draw heatmap. (**D**) Venn diagrams illustrating the relationships among DEGs from different comparisons. (**E**) KEGG enrichment profiles of upregulated DEGs detected in the pairwise comparisons.

RNA-seq analysis identified 19,940 unique differentially expressed genes when samples collected at 6, 24, 72, and 120 h were compared with the 0 h uninfected control. A total of 6796 DEGs between 0 h and 6 h (4298 genes upregulated in 6 h, 2498 genes upregulated in 0 h); 12,637 DEGs between 0 h and 24 h (9491 genes upregulated in 24 h, 3146 genes upregulated in 0 h); 8059 DEGs between 0 h and 72 h (5444 genes upregulated in 72 h, 2615 genes upregulated in 0 h); and 12,224 DEGs between 0 h and 120 h (7037 genes upregulated in 120 h, 5187 genes upregulated in 0 h) (Figure 1A). The 0 h/24 h and 0 h/120 h comparisons contained more DEGs than the other two comparisons: the 0–6 h and 0–72 h intervals.

According to KEGG enrichment, genes associated with plant hormone signal transduction and the MAPK signaling cascade underwent dramatic transcriptional changes during the infection (Figure 1B). Plant–pathogen interaction-related genes also dramatically changed during the infection. Meanwhile, genes involved in biosynthetic pathways like brassinosteroid biosynthesis and diterpenoid biosynthesis and pathways related to carotenoid production, flavonoid formation, phenylpropanoid synthesis, and the formation of unsaturated fatty acids were also differentially regulated during the course of infection.

GO analysis showed that cell-wall-related terms, like plant-type cell-wall organization, plant-type cell-wall modification involved in multidimensional cell growth, plant-type cell-wall cellulose metabolic process, and plant-type secondary cell-wall biogenesis-related genes, dramatically changed during infection (Figure 1C). JA and ethylene-dependent systemic resistance, calcium-mediated signaling, positive regulation of systemic acquired resistance, and GO terms related to symbiont-induced activation of host innate immune responses via pathogen-associated molecular patterns (PAMPs) were found to be specifically enriched in the 0 h vs. 6 h comparison, indicating their potential involvement in mediating host defenses against the infection. Gene expression, positive regulation of wound healing, positive regulation of cell differentiation, and negative regulation of stress-activated MAPK cascade-related GO terms were specifically enriched in the 0 h/72 h comparison. The cornification and negative regulation of oxidoreductase activity-related GO terms were specifically enriched in 0 h/120 h comparison. Anion channel activity, nucleoside transmembrane transporter activity, and hydrogen peroxide-induced cell death, defense response, cell wall, and response to plant hormone, especially JA and SA, were persistently enriched throughout the entire infection process. These results suggested that different biologic processes occurred in different stages of infection. Phytohormone-mediated signal transduction, defense responses, and the plant-specific MAPK cascade played pivotal roles during *C. pilosula* defense against *F. oxysporum* attack. We also identified the DEGs specifically expressed in each comparison (Figure 1D). A total of 324, 3936, 544, and 2358 genes were upregulated at 6, 24, 72, and 120 h compared with 0 h, respectively. Functional analysis of these genes showed that phenylalanine, tyrosine and tryptophan biosynthesis, propanoate metabolism, and Glycolysis/Gluconeogenesis-related pathways were enriched in 6 h compared with 0 h. Peroxisome and Proteasome-related genes were upregulated in 24 h compared with 0 h. Basal transcription factors and genes associated with MAPK cascades and plant–pathogen defense systems exhibited elevated expression at 72 h compared with 0 h. Cutin, suberine, and wax biosynthesis, biotin metabolism, and oxidative phosphorylation-related pathways were highly enriched in 120 h compared with 0 h (Figure 1E). Therefore, different biologic processes occurred in different stages of infection.

### 3.3. K-Means Cluster Revealed the Response of C. pilosula to F. oxysporum

**Figure 2:**
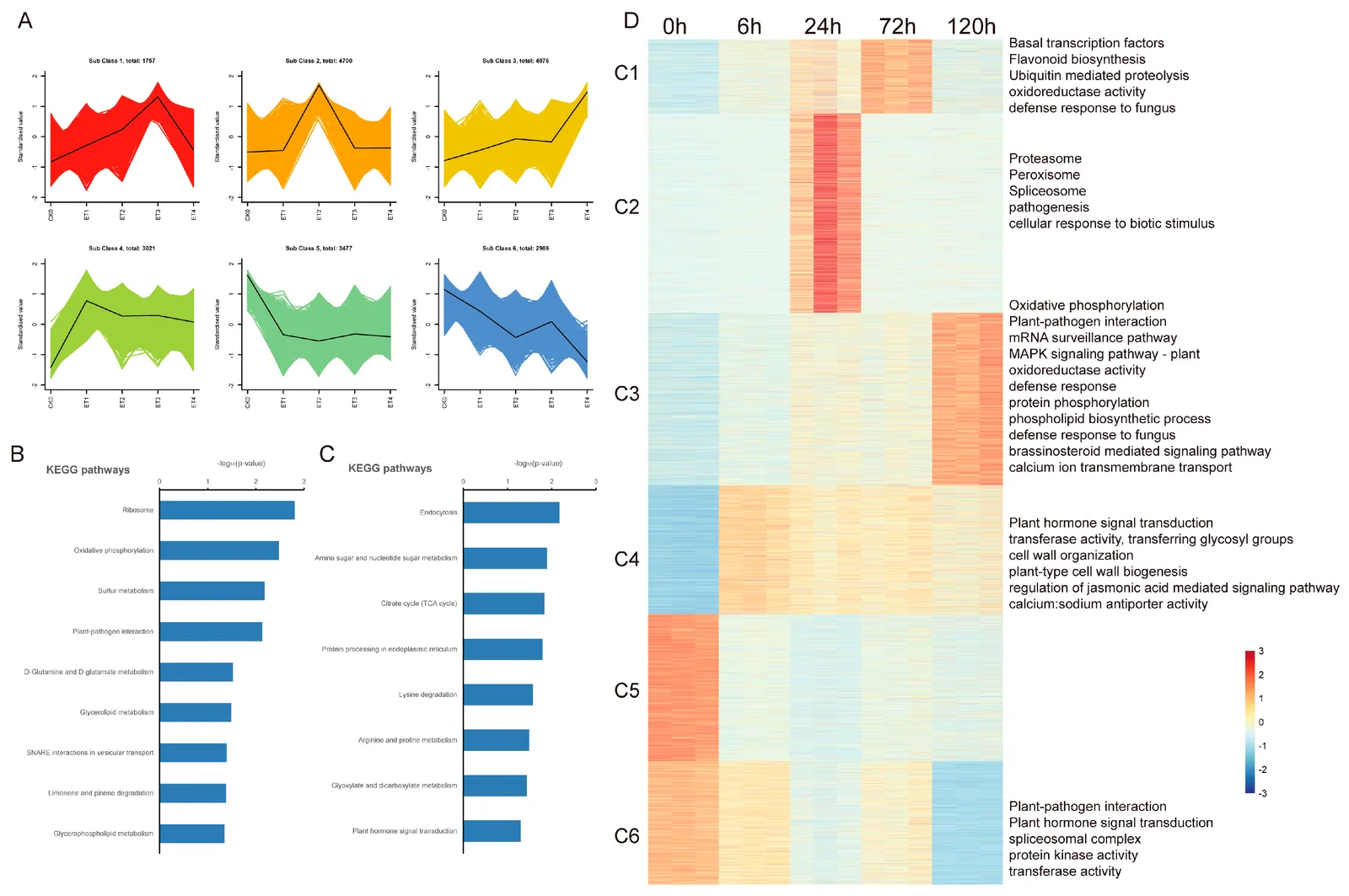
K-means clustering and functional analysis of DEGs. (**A**) A total of 6 clusters of DEGs based on FPKM values. KEGG enrichment of the DEGs in class 3 (**B**) and class 4 (**C**). (**D**) Expression profiles and functional characteristics of DEGs. Z-score values of normalized FPKM for each gene were used to draw a heatmap.

We further explored the expression pattern of the DEGs by k-means cluster analysis and generated a total of six clusters. As shown in Figure 2A, genes involved in class 3 gradually increased after infection. Meanwhile, genes involved in class 4 increased after the infection, peaked at 6 h, and then decreased. Genes involved in class 1 and class 2 gradually increased and peaked at 72 and 24 h, respectively. Genes clustered into class 6 decreased after infection, and a brief uptick occurred within 72 h before the decline continued. Class 5 genes showed an obvious decrease after infection.

KEGG analysis showed that class 3 genes exhibited significant enrichment within the “plant–pathogen interaction” term, whereas those assigned to class 4 were mainly associated with the plant hormone signal-transduction pathway (Figure 2B,C). We explored the expression pattern and functional characteristics of the DEGs, as shown in the heatmap (Figure 2D). The expression of genes (C3) enriched in the plant–pathogen interaction, MAPK signaling pathway, and defense response pathways gradually increased during infection. For example, several calcium-dependent protein kinase genes were highly expressed within 120 h after infection. Disease-resistance proteins like RF45, RF5, and RPM1 were highly expressed within 120 h. Numerous WRKY transcription factors also showed high expression levels during the later stage of infection. Meanwhile, genes (C3) involved in oxidoreductase activity, protein phosphorylation, the brassinosteroid-mediated signaling pathway, and calcium-ion transmembrane transport also exhibited elevated expression levels during the later stage of infection. Three brassinosteroid insensitive 1-associated receptor kinase were also highly expressed in the later stage of infection. Genes (C4) associated with phytohormone signal transduction showed high expression in the early stage of infection. Cell-wall organization, plant-type cell-wall biogenesis, regulation of JA-mediated signaling pathway, and calcium/sodium antiporter activity-related genes also exhibited elevated expression levels at 6 h after infection. These findings suggested that these genes may be the first to respond to infection.

Flavonoid biosynthesis, Ubiquitin-mediated proteolysis, oxidoreductase activity, and defense response to fungus-related genes gradually increased after infection. Meanwhile, basal transcription factors like transcription initiation factor IIA/B also gradually increased after infection, peaking at 72 h. Proteasome, Peroxisome, Spliceosome, pathogenesis, and cellular response to biotic stimulus-related genes (C2) showed elevated expression at 24 h. In cluster C6, genes involved in plant–pathogen interaction, plant hormone signal transduction, spliceosomal complex, protein kinase activity, and transferase activity were also highly expressed at 6 and 72 h. This finding indicated that plant hormones and plant–pathogen interaction-related genes were the earliest network of plant response to infection.

### 3.4. Involvement of Hormones in the Defense Mechanisms of C. pilosula

**Figure 3:**
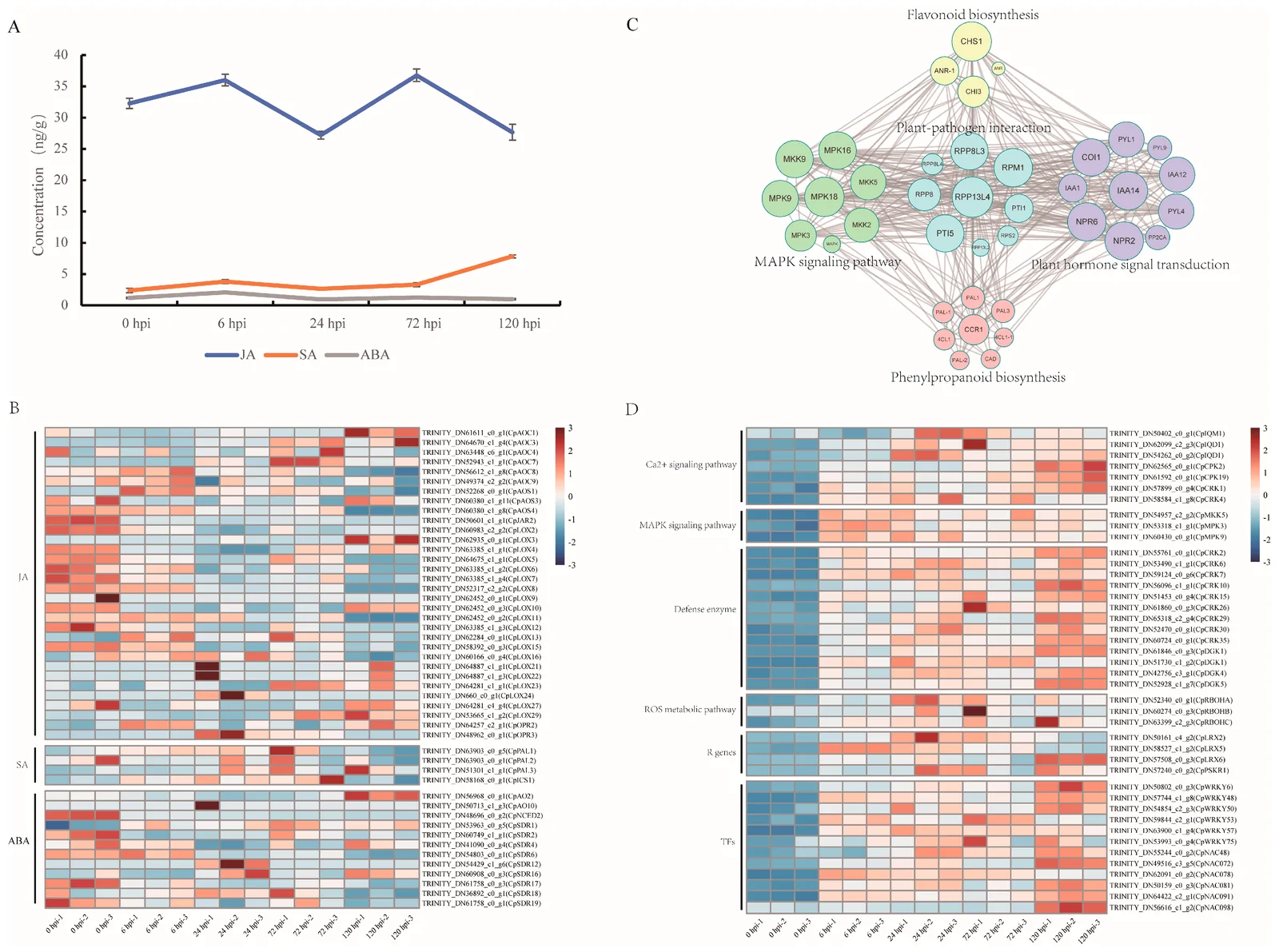
Hormone and related genes change after infection with *Fusarium oxysporum*. (**A**) Content of JA, SA and ABA during infection. (**B**) Expression profiles of genes involved in JA, SA, and ABA biosynthesis. (**C**) Interaction network of genes involved in infection. (**D**) Heatmap of genes continuously upregulated in response to infection. The bar represents the scale of the expression levels for each gene (FPKM) at different time points.

Following inoculation with *F. oxysporum*, genes associated with the JA, SA, and ABA signaling pathways were significantly induced. To investigate the roles of JA, SA, and ABA in the defense response against *F. oxysporum*, we quantified the levels of these hormones in *C. pilosula* at designated intervals (0, 6, 24, 72, and 120 h) following inoculation (Figure 3A). Results showed that the contents of JA, SA, and ABA began to increase as early as 6 h after treatment, with SA levels peaking at 120 h. These findings suggested that JA, SA, and ABA played critical and temporally distinct roles in the defense mechanisms of *C. pilosula* against *F. oxysporum*.

Further analysis revealed that key genes associated with JA signaling, such as lipoxygenases (CpLOX23 and CpLOX29) and allene oxide cyclases (*CpAOC1*, *CpAOC3*, and *CpAOC7*), were highly expressed (Figure 3B). Similarly, genes involved in SA signaling such as phenylalanine ammonia-lyases (*CpPAL1* and *CpPAL3*) and isochorismate synthase (*CpICS1*) were significantly upregulated. Additionally, genes in the ABA signaling pathway, including abscisic aldehyde oxidase (*CpAO2*) and short-chain dehydrogenase/reductase (*CpSDR1*), exhibited high expression levels. These results collectively highlighted the activation of JA, SA, and ABA signaling pathways as a coordinated response to *F. oxysporum* infection in *C. pilosula*.

To explore the molecular mechanisms involved in disease resistance, we constructed and analyzed a gene interaction network (Figure 3C). The results showed extensive interactions among genes in the MAPK signaling pathway, plant hormone signaling pathway, flavonoid biosynthesis pathway, phenylpropanoid biosynthesis pathway, and plant–pathogen interaction pathway. Notably, key genes such as *MAPK9*, *CHS1*, *RPP13L4*, *IAA14*, and *CCR1* exhibited prominent interactions across these pathways, suggesting their potential central roles in mediating the defense response. These findings indicated that these genes may play a critical role in the resistance of *C. pilosula* to root rot caused by *F. oxysporum*, highlighting their importance in the plant’s defense mechanisms.

To further elucidate the key DEGs associated with the resistance of *C. pilosula* to *F. oxysporum*, a heatmap was constructed (Figure 3D). The heatmap indicated a pronounced upregulation of genes related to Ca^2+^ signaling, as well as those involved in MAPK signaling, defense enzymes, reactive oxygen species (ROS), R genes, and transcription factors following *F. oxysporum* inoculation. The results demonstrate that these pathways and gene categories contribute significantly to *C. pilosula* resistance against *F. oxysporum* infection and may play important roles in the associated defense mechanisms.

### 3.5. Identification of CpMAPKs and the Role of CpMAPK9 in Regulating JA Biosynthesis to Promote Disease Resistance 

To better understand the mechanism of *C. pilosula* root rot, we investigated the *CpMAPK* genes. Based on *C. pilosula* transcriptome data, a total of 10 *CpMAPK* genes were identified by BLASTP (v2.13.0) comparison. Physicochemical analysis showed that the length of the *CpMAPK* gene ranged from 1658 bp to 2592 bp, and the length of the open reading frame ranged from 1657 bp to 2591 bp. The encoded proteins varied in length from 234 to 482 bp, with molecular weights ranging between 27,060.13 and 56,918.83 Da and isoelectric points ranging between 5.49 and 9.19. The grand average of hydropathicity (GRAVY) values ranged from −0.519 to −0.152, all of which were negative, suggesting that the CpMAPK proteins are hydrophilic. Subcellular localization analysis predicted that all CpMAPK proteins were localized in the cytoplasm. Detailed genetic information is provided in Supplemental Table S2.

**Figure 4:**
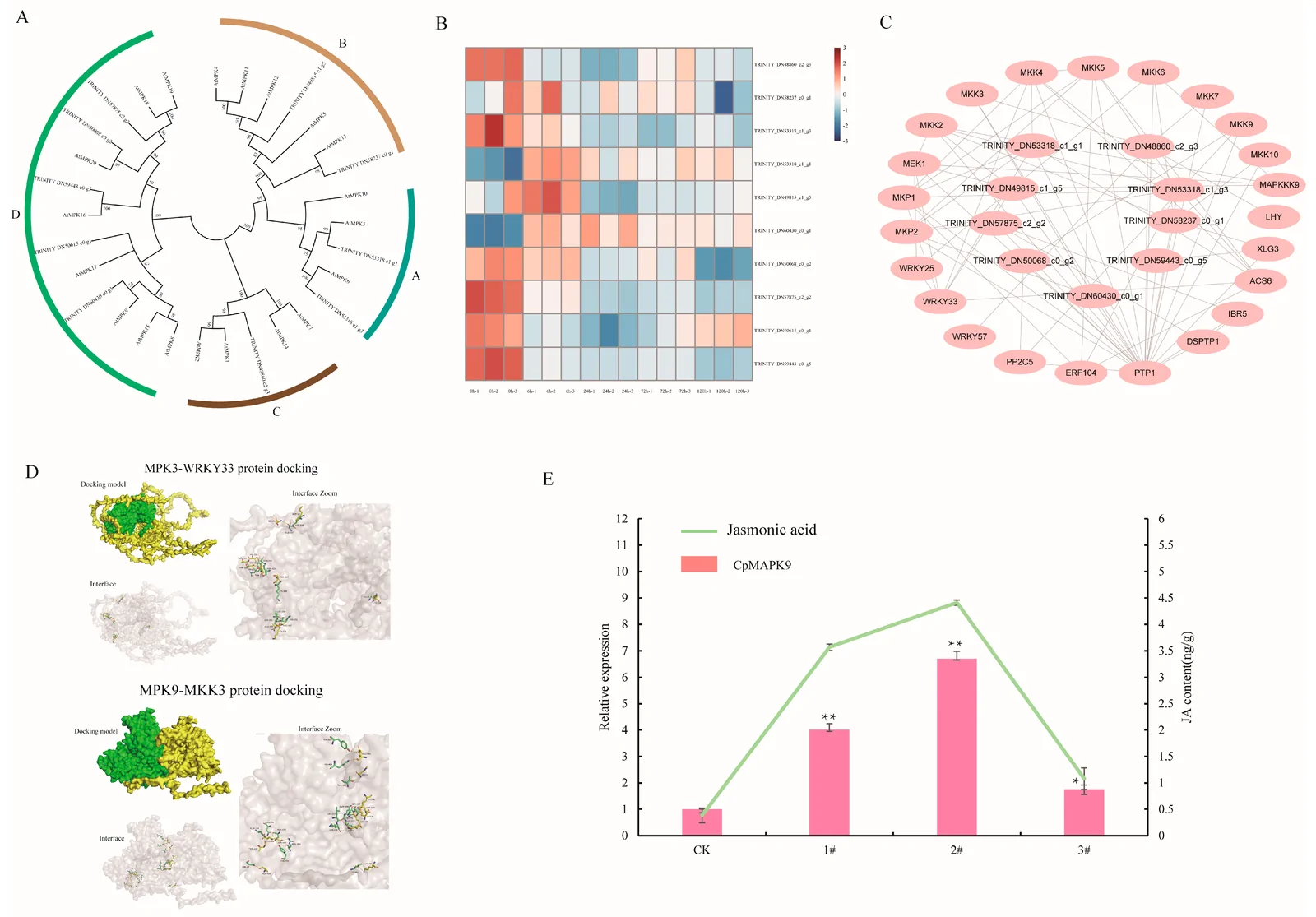
MAPK family analysis and *MAPK9* function analysis. (**A**) Phylogenetic evolutionary tree of the *C. pilosula* MAPK family. Triangulum is a member of the *A. thaliana* MAPK family. The green triangle is subgroup A, the purple triangle is subgroup B, the blue triangle is subgroup C, and the red triangle is subgroup D. (**B**) Analysis of conservative motif of MAPK of *C. pilosula*. (**C**) Interaction network of MAPK proteins. The inner 9 oval markers are CpMAPK proteins, and the outer 23 oval markers are interacting proteins. (**D**) MPK3 and WRKY33 protein docking. MPK9 and MKK3 protein docking. (**E**) Relative *CpMAPK9* expression and JA content in WT and three OE lines. Values are expressed as mean ± SD (*n* = 3). Asterisks indicate significant differences determined by one-way ANOVA (*p* < 0.01). * *p* < 0.05 and ** *p* < 0.01 indicate significant differences determined by one-way ANOVA.

The phylogenetic evolutionary tree was constructed based on the amino acid sequences of the MAPK family of *C. pilosula* and the MAPK family of *A. thaliana*, and the evolutionary relationships of CpMAPK family members were analyzed (Figure 4A). The phylogenetic evolutionary tree showed that CpMAPK family members are classified into four subfamilies: A, B, C, and D. Subgroup A contained two CpMAPKs, subgroup B contained two, and subgroup C contained one. Subgroup D contained five (the largest number of CpMAPKs), accounting for 50% of the total.

To investigate the response of the *CpMAPK* gene to biotic stress, its expression levels were evaluated using FPKM values at 0, 6, 24, 72, and 120 h following *F. oxysporum* infection of *C. pilosula* (Figure 4B). Results showed that the expression levels of TRINITY_DN53318_c1_g1 and TRINITY_DN60430_c0_g1 increased with increased infection time, whereas the expression levels of other genes decreased with increased infection time. Therefore, we speculated that TRINITY_DN53318_c1_g1 and TRINITY_DN60430_c0_g1 may play an important role in mediating how *C. pilosula* defends against *F. oxysporum*.

To gain deeper insights into the prospective physiological functions as well as regulatory networks of MAPK genes in *C. pilosula*, possible protein–protein interaction networks were predicted based on homology analysis. Nine CpMAPK proteins homologous to *A. thaliana* and 23 proteins interacting with them were identified (Figure 4C). The proteins that interacted with CpMAPK were MAPKKs (MEK1, MKK2, MKK3, MKK4, MKK5, MKK6, MKK7, MKK9, and MKK10) and MAPKKK9. CpMAPK also interacted with two MAPK phosphatases (MKP1 and MKP2). CpMAPK proteins interacted with WRKY25, WRKY33, and WRKY57 (Figure 4D). Additionally, CpMAPK proteins interacted with PP2C5, ERF, and PTP proteins. These proteins and CpMAPK proteins may jointly respond to *F. oxysporum* infection, thereby regulating the defense response after *F. oxysporum* infection. This factor might also critically modulate how MAPK proteins function in *C. pilosula*.

To further elucidate the functional significance of *CpMAPK9*, we transiently overexpressed this gene in the leaves of *C. pilosula*. Compared to the empty-vector-carrying WT plants, the OE lines exhibited a pronounced accumulation of JA (Figure 4E). These findings indicated that *CpMAPK9* may play a regulatory role in JA biosynthesis, potentially enhancing disease resistance in *C. pilosula*. These findings provided insights into the molecular mechanisms by which *CpMAPK9* contributed to the plant’s defense response against pathogen infection.

## 4. Discussion

The development of the *C. pilosula* industry is currently threatened by root rot, a serious disease primarily associated with *F. oxysporum* infection. This disease infects the roots of *C. pilosula*, leading to root rot and necrosis, which severely reduces plant productivity and quality and causes considerable financial losses. Therefore, in-depth research into the mechanisms of *C. pilosula* resistance to *F. oxysporum* has great importance for developing effective disease-control strategies and breeding resistant varieties. When pathogens breach the mechanical barriers of a plant, the innate immune system is activated to limit microbial colonization and invasion [21,22]. In the case of *C. pilosula* infected by *Fusarium o.*, changes in enzyme activity were observed as part of the plant’s defense response to counteract pathogen invasion. RNA-seq analysis was used to identify DEGs at specific time points during infection. The substantial number of detected DEGs indicated that *C. pilosula* underwent significant transcriptional reprogramming in response to pathogen stress, reflecting a robust regulatory network for biotic stress resistance. This network likely enhanced the plant’s ability to perceive and transduce stress signals effectively.

Notably, metabolic pathways such as flavonoid and phenylpropanoid biosynthesis were recurrently upregulated upon infection [7], suggesting that *C. pilosula* could produce secondary metabolites to inhibit pathogen progression [23,24]. Furthermore, genes associated with the plant hormone signal-transduction pathway and the MAPK signaling pathway were significantly enriched during *F. oxysporum* infection (Figure 1). These findings highlighted the critical roles of hormone signaling and MAPK cascades in coordinating the defense response, thereby underscoring the complexity and efficiency of *C. pilosula*’s immune mechanisms against pathogen stress.

By phosphorylating diverse transcription factors—including the WRKY, ERF, and MYB families—MAPK cascades serve as crucial mediators in conveying biotic stress signals [25]. Specifically, MAPK cascades primarily target WRKY transcription factors. For example, the MPK3/MPK6-WRKY33 module was involved in signaling during *Botrytis cinerea* infection, whereas MAPK-WRKY7/8/9/11 pathways are implicated in responses to *Phytophthora infestans* [26,27]. In *C. pilosula*, CpMAPK was found to interact with WRKY transcription factors, suggesting that CpMAPK may undergo stepwise phosphorylation mediated by MAPKs to regulate WRKYs and other transcription factors, thereby playing a critical role in resistance to root rot pathogens. MAPKs reportedly function in plant immunity, either positively or negatively modulating disease signaling [28,29]. The regulation of ethylene and camalexin biosynthesis by the MKK4/5-MPK3 cascade during *Botrytis cinerea* infection highlights its critical involvement in both fungal and bacterial defense pathways [30,31].

In this study, a total of 10 *CpMAPK* genes were identified from *C. pilosula* transcriptome data. Spatiotemporal differences in *MAPK* gene expression have been reported in various plants, such as *Cordyceps* [32], melon [33], tea tree [34], and barley [35]. Similarly, our study revealed time-dependent expression patterns of *CpMAPK* genes in *C. pilosula* under *F. oxysporum* infection, with *CpMAPK9* showing particularly significant expression levels. In addition, we have found that *CpMAPK9* could improve the disease resistance of plants by enhancing the accumulation of SA and JA [36,37,38,39]. In *C. pilosula*, SA and JA signaling genes were highly active during *F. oxysporum* infection (Figure 4), leading to increased synthesis and signaling of these hormones to initiate defense processes. In particular, JA has been reported to regulate the transcription, protein accumulation, and the kinase activity of MAPK signaling components in various plants [40]. Consistent with this finding, we found that *CpMAPK9* OE resulted in elevated JA levels, suggesting a potential synergistic relationship between *CpMAPK9* and JA in enhancing disease resistance in *C. pilosula.* Nonetheless, our current evidence (transient overexpression and JA accumulation) does not fully establish a causal link between *CpMAPK9* and root rot resistance, and additional phenotypic and genetic evidence is needed to further validate this association. To address these limitations, our upcoming research will focus on optimizing the transformation protocol and confirming phenotypic uniformity among multiple independent transgenic hairy-root lines, thereby providing more robust genetic validation. These findings highlighted the importance of MAPK cascades and hormone signaling pathways in coordinating the plant’s immune response to pathogen stress.

## 5. Conclusions

In our study, based on the transcriptome data, gene family analysis, and functional analysis of key genes, the relevant resistance pathways, plant hormone signaling networks, and MAPK signal-transduction pathways of *C. pilosula* were found to be involved in the response to *F. oxysporum*. Then, the MAPK family was analyzed to clarify how *C. pilosula* responds to *F. oxysporum* infection. Through MAPK pathway enrichment and systematic examination of MAPK family genes, the *CpMAPK9* gene was mined and identified. Its function was explored, and the results suggested a potential role in enhancing resistance to *C. pilosula* root rot, possibly through the jasmonic acid signaling pathway based on transcriptomic evidence. However, the direct involvement of the JA pathway requires further experimental validation. This work establishes a theoretical framework for developing *C. pilosula* cultivars with enhanced resistance to *F. oxysporum*.

## Figures and Tables

**Table 1:** Summary of the *C. pilosula* transcriptome data.

Sample	0 h-1	0 h-2	0 h-3	6 h-1	6 h-2	6 h-3	24 h-1	24 h-2	24 h-3	72 h-1	72 h-2	72 h-3	120 h-1	120 h-2	120 h-3
Raw_Reads	38,659,818	45,984,576	53,563,676	48,686,672	56,218,658	45,108,692	50,154,916	38,371,478	48,552,570	45,166,426	40,514,964	37,965,234	34,084,348	43,374,460	42,104,862
Valid_Reads	38,158,716	45,412,206	52,821,412	48,210,882	55,653,308	44,628,990	49,473,952	37,877,678	47,948,230	44,496,236	39,777,702	36,840,720	33,288,528	42,526,154	41,184,208
Valid%	98.70	98.76	98.61	99.02	98.99	98.94	98.64	98.71	98.76	98.52	98.18	97.04	97.67	98.04	97.81
Q20%	98.11	98.19	98.10	98.29	98.23	98.20	98.13	98.22	98.21	98.12	98.06	97.88	97.84	98.00	97.93
Q30%	94.04	94.20	94.02	94.40	94.26	94.21	94.15	94.26	94.24	94.08	93.96	93.71	93.69	93.91	93.86
GC%	44.80	44.82	44.98	44.68	44.65	44.74	45.16	45.15	44.75	44.90	44.80	45.11	44.98	44.92	44.80
Gene Number	94,896														
GC%	42.80														
Min Length	201														
Max Length	14,715														
Total Assembled Bases	74,754,051														
N50	1435														

## Data Availability

These data are not publicly available due to privacy and confidentiality considerations, as well as their ongoing use in collaborative research projects. The raw data supporting the conclusions of this article are available on request from the corresponding author.

## Supplementary Materials

The following supporting information can be downloaded at https://www.mdpi.com/article/10.3390/genes17080890/s1: Supplementary Figure S1. Cloning of the *CpMAPK9* gene; Supplementary Figure S2. Changes in physiological indexes of *C. pilosula* during *F. oxysporum* infection. (A–D) Change of root rot at 0, 6, 24, 72 and 120 hours after *F. oxysporum* infection. (E) PCA analysis among different samples. (F) Correlation analysis among different samples; Supplementary Figure S3. The qPCR data for the six DEGs; Supplementary Table S1. Genes and their primer sequesnces for qRT-PCR; Supplementary Table S2. Summary of the *CpMAPK* gene information data.
