# Supplementary material for: High Expression of CpMAPK9 Increased Jasmonic Acid Levels Potentially Improving Root Rot Resistance in Codonopsis pilosula
Source: Genes (Basel). 2026 Jul 29;17(8):890. doi: 10.3390/genes17080890 (PMC13512866; doi:10.3390/genes17080890)

Supplementary Figure

Figure S1 Cloning of the CpMAPK9 gene.

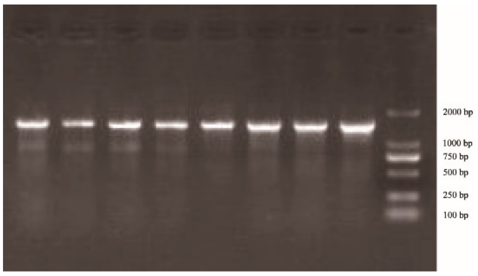

Figure S2 Changes of physiological indexes of C. pilosula during F. oxysporum infection. **A-D**. Change of root rot at 0, 6, 24, 72 and 120 hours after F. oxysporum infection. **E**. PCA analysis among different samples. **F.** Correlation analysis among different samples.

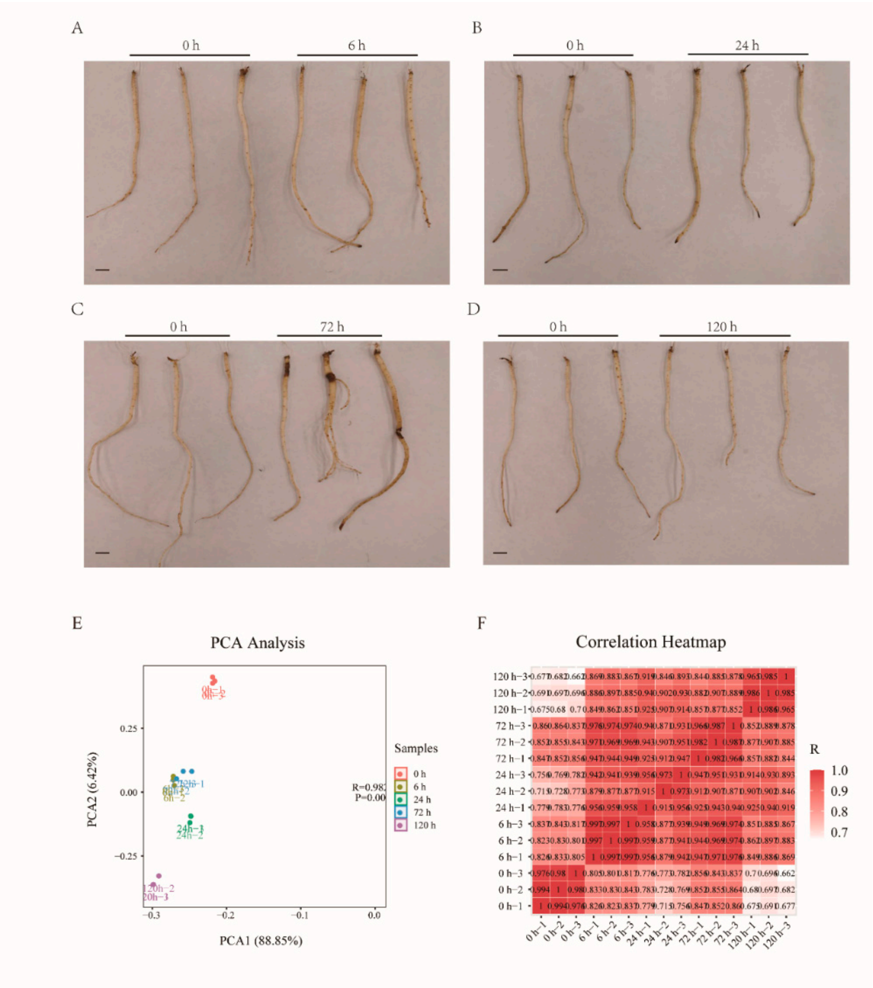

Figure S3 The qPCR data for the six DEGs.

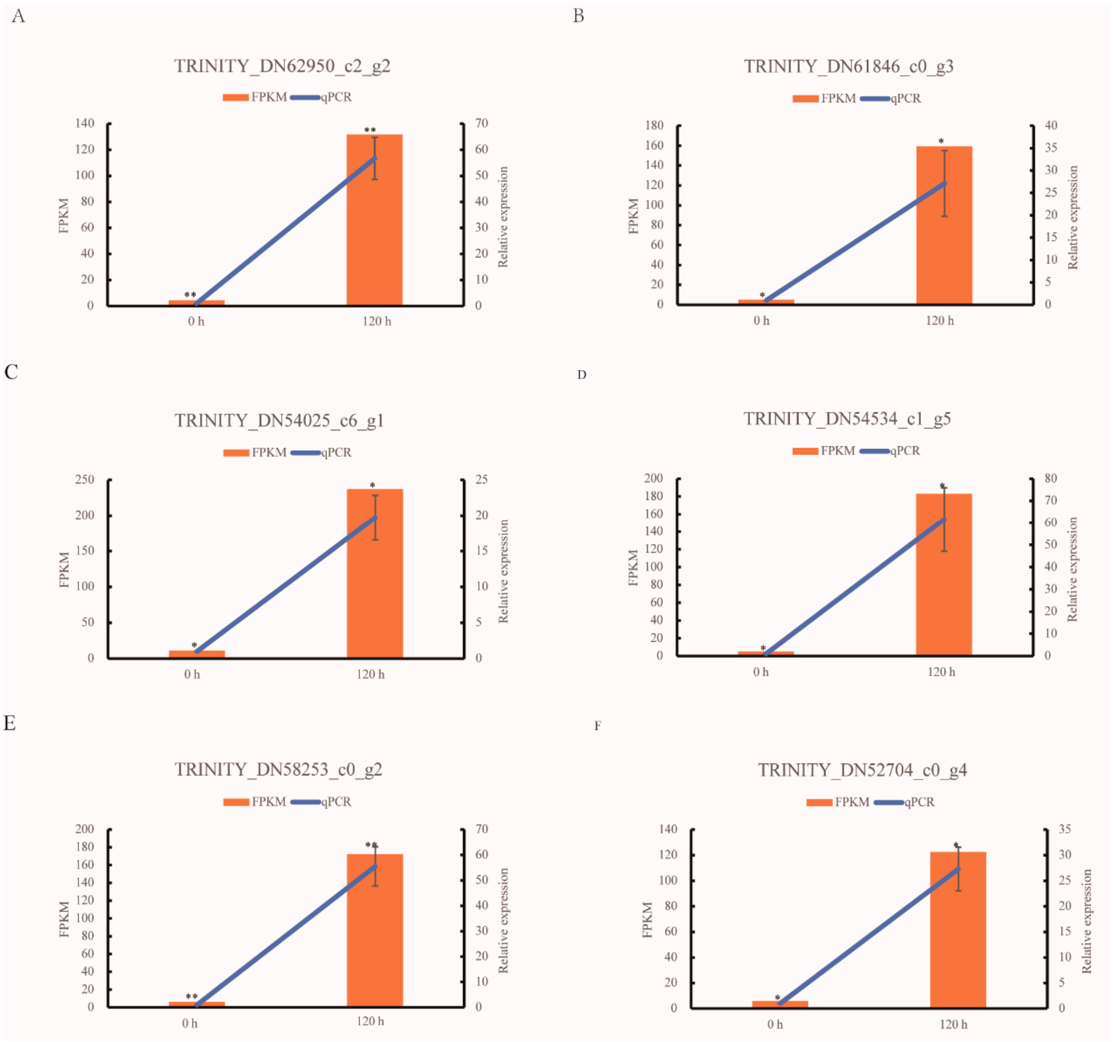

Supplement: Supplementary file 1 [file genes-17-00890-s001.zip › Supplementary date.pdf]
